# Evaluation of the National Cancer Institute (NCI) Pathway to Independence Award (K99/R00) Program

**DOI:** 10.1007/s13187-024-02420-1

**Published:** 2024-03-18

**Authors:** Michael Schmidt, Corinne Boulanger-Espeut, Grace Liou, Nan Ma, Sasha Torres, Susan Cersosimo, Oliver Bogler, Nastaran Zahir

**Affiliations:** 1 National Cancer Institute, National Institutes of Health, Rockville, MD 20850 USA; 2 Digital Science, Inc, Cambridge, MA 02139 USA

**Keywords:** Career development, NIH grants, K99/R00

## Abstract

The National Cancer Institute (NCI) K99/R00 award is intended to help postdoctoral scholars transition in a timely manner to research independence and to foster their development of an impactful cancer research program that is competitive for subsequent independent funding. Here we analyzed factors that impact peer review outcomes and evaluated whether NCI K99/R00 awardees have achieved the goals of the K99/R00 funding mechanism. Our analysis of the K99/R00 review criterion scores demonstrates that while all review criterion scores are positively correlated with the overall impact score, the Research Plan criterion is the strongest predictor of the overall impact score and funding outcomes. In addition, our analysis shows the NCI K99/R00 award facilitated the successful transition of postdoctoral scholars to research independence and enhanced the likelihood of K99/R00 awardees to secure subsequent R01-equivalent NIH grant support although not in an accelerated fashion as originally intended. An NCI K99/R00 award was not determined to be a prerequisite to obtain a faculty position, but for some awardees, it was an asset in that transition. Our results suggest that the NCI K99/R00 award is an important component for training and retention of the next generation of independent cancer researchers and to increasing the percentage of women and promoting the diversity of the cancer research workforce.

## Introduction

The mission of the National Cancer Institute (NCI) Center for Cancer Training (CCT) is to catalyze the development of a twenty-first century workforce capable of advancing cancer research through a scientifically integrated approach. The NCI is constantly identifying and evaluating workforce needs in cancer research and adapting training, career development programs, and funding opportunities to address these needs. The NCI CCT Cancer Training Branch (CTB) supports training and education opportunities across all cancer research disciplines, allowing early-stage cancer researchers to acquire skills and resources for career advancement toward research independence. The NCI individual Research Career Development (K) Awards are an important tool for cancer workforce development and are intended to ensure that a diverse pool of highly trained scientists is available to address the Nation’s biomedical, behavioral, and clinical cancer research needs.

Individual K awards are designed to provide research training and career development opportunities for early career stage investigators through mentorship, salary and research support, and protected time for research. One such award is the K99/R00 Pathway to Independence Award, which was launched by the National Institutes of Health (NIH) in 2006 (https://grants.nih.gov/policy/early-stage/history). The K99/R00 was established to help postdoctoral researchers complete needed, mentored career development, transition in a timely manner to independent, tenure-track or equivalent faculty positions and develop a creative, independent research program that will be competitive for subsequent independent funding. This award provides up to 2 years of mentored postdoctoral career development (K99 phase) followed up by up to 3 years of independent research support (R00 phase) at a level similar to an R01. The transition to the R00 phase is contingent upon satisfactory progress during the K99 phase and securing a competitive independent, tenure-track (or equivalent) faculty position.

Competition for these early career researchers to secure postdoctoral and subsequent faculty positions has been strong, with many qualified candidates seeking to fill a limited number of available jobs. To acquire research funding after attaining tenure-track (or equivalent) positions, researchers must focus on being scientifically productive while also publishing their work. The competition for funding is also stringent, resulting in a trend toward an increased age at which researchers achieve independence (https://go.nih.gov/FMBbhEL) as determined by obtaining of the first NIH research project grant (RPG) (https://grants.nih.gov/grants/glossary.htm#ResearchProjectGrant(RPG)). This raises several concerns. First, the academic career path may appear risky and less attractive to some early career investigators, both because there is no guarantee of a faculty position after years of postdoctoral research and beyond that, the fact that it can take 4–5 years [1] to establish oneself as an independent researcher after being hired. Second, creativity and innovations are thought to decline as an investigator increases in age [2, 3]. For this reason, the NIH created the K99/R00 award, which was designed to accelerate the transition to independent research and make awardees competitive for faculty positions. The R00 phase was intended to make awardees more attractive to hiring organizations as it both demonstrates the awardees’ ability to obtain NIH funding and brings 3 years of funds at a research project grant level including indirect costs at the current negotiated R00 institution’s facilities & administrative (F&A) rate. Furthermore, because the K99/R00 award requires protected time for research, it allows the principal investigator to immediately focus on building their research team and program and maximize their research productivity, also with the help of the R00 funds. We investigated the outcomes of NCI’s K99/R00 to understand whether they matched the design and intent of the program.

The NCI issued its first K99/R00 awards in 2007, and at its inception, eligibility allowed for up to 5 years of postdoctoral research experience for qualifying applicants in mentored, non-independent positions. In 2014, eligibility was reduced to 4 years of postdoctoral research experience with the goal of further accelerating the time to research independence for NIH Early Stage Investigators (ESIs). In initial years, areas of research supported by the K99/R00 were limited to laboratory-based cancer research, with population, cancer prevention, control, and behavioral sciences being referred to the NCI K07 or K22 funding mechanisms. In 2015, the NCI K99/R00 began accepting applications in all areas of cancer research. While the K99/R00 award could support physician-scientists who already have substantial research training, the eligibility window is a hindrance for individuals who need a longer period of mentored career development. Therefore, physician-scientists are typically encouraged to consider the K08 Clinical Scientist Research Career Development Award.

The K99/R00 quickly became one of the NCI’s most popular career development funding opportunities averaging 43% of all competing K-applications and 35% of all competing K-awards from fiscal year (FY) FY2018–FY2022. The interest and investment in the NCI K99/R00 align with the mission of the NCI to ensure the training and development of a diverse pool of highly skilled scientists in a wide variety of scientific disciplines that can meet the needs of the nation’s biomedical, behavioral, and clinical cancer research needs. Here, we examine whether the NCI K99/R00 has had the intended positive impact on the careers of early career cancer investigators.

## Methods

### Cohorts

- Study group: NCI K99/R00 awardees from FY2007 to FY2017.
- Comparison group 1: F32 awardees from FY2005 to FY2015.
- Comparison group 2: NCI K99/R00 applicants from FY2007 to FY2017 who did not receive an NCI K99/R00 award. We divided this group into two cohorts: (a) NCI K99/R00 applicants whose applications were discussed and received a score during the peer review meeting (K99_dis); and (b) NCI K99/R00 applicants whose applications were not discussed and so received the ND designation (K99_ND). The K99/R00 cohorts were exclusive (i.e., each PI was only assigned to one cohort), but 4.5% of F32 awardees also received an NCI K99/R00 award between FY2007 and FY2017 and were included in both the K99 awardee and F32 awardee cohorts.

Data for the study and comparison cohorts were extracted from the NIH Information for Management, Planning, Analysis and Coordination II (IMPACII) database. Demographics data for K99/R00 applicants including race, ethnicity, sex/gender, and age data self-identified in eRA Commons were obtained via a Data Use and Data Access agreement from the NIH Office of Extramural Research. Demographics data are only shown if the aggregated total in any group is ≥ 12. The annual success rate was calculated as the percentage of reviewed grant applications that were awarded. Applications having one or more submissions for the same project in the same FY were only counted once (https://report.nih.gov/funding/nih-budget-and-spending-data-past-fiscal-years/success-rates). The award rate was used for multi-year studies and was calculated by determining the percentage of reviewed grant applications that were awarded (this included applications having one or more submissions for the same project in the same FY).

We analyzed whether K99/R00 and F32 applicants submitted and received subsequent NIH Research Project Grants (RPG) or R01-equivalent (R01eq) grants (https://grants.nih.gov/grants/glossary.htm#R01equivalentGrant) and the number of R01eq awards. In addition, we examined the time (in years) between the applicants’ PhD graduation and submission or award of their first RPG or R01eq grant application. Data from applicants without a PhD degree, e.g., applicants with a medical degree only (0.1% of applicants), were excluded from the analysis because candidates with a medical degree have a career development timeline that is different from that of candidates with PhDs.

### Data Analysis

For the criterion score analysis, we extracted the overall impact score and reviewers’ criterion scores for K99/R00 applications from FY2010 to FY2017. The criterion scores were calculated by averaging the individual criterion scores given by the reviewers for each application. Criterion scores were introduced in FY2010 as part of the NIH’s Enhancing Peer Review effort (https://grants.nih.gov/grants/guide/notice-files/NOT-OD-09-023.html).

Descriptive summary statistics, correlations between the five criterion scores and the overall impact score and linear regression analysis was performed in Excel after averaging the individual reviewer criterion scores.

### Survey

We surveyed K99/R00 recipients (*n* = 352) who received the award between FY2007 and FY2017 and NCI ESI Principal Investigators (PI’s) who received an R01 or R37 award between FY2017 and FY2022 but never applied for an NIH K99/R00 (non-K99/R00 recipient, *n* = 538). The subjects were sent an email containing the Survey Monkey tool using the email addresses they reported in their NIH eRA Commons account. In the email, we indicated an estimated time of 1 min to complete the 4-question survey and we provided 10 days for completion of the survey. We recognize that email addresses may be outdated if subjects had not updated their NIH eRA Commons account. There was no follow-up to the initial request for responses because the response rate was sufficient for our evaluation. We obtained complete responses from 193 K99/R00 recipients and 323 non-K99/R00 recipients. We then randomly selected a group of 21 K99/R00 recipients and 19 non-K99/R00 recipients who volunteered for an informal follow-up 10–15-min phone interview.

For K99/R00 recipients, we asked if the K99/R00 award helped them compete for a position as an independent researcher with choices of yes, no, or I don’t know. We then asked a follow-up question of why they applied for the K99/R00 award. The respondent could choose to indicate that it was due to a recommendation from a mentor and/or a peer or provide a free-text personal response. Twenty-one of these respondents, who also indicated a willingness to be interviewed, were then randomly chosen for a follow-up phone interview to provide further detail on the reasons why they chose to apply for the award, and its impact on their career trajectory.

The non-K99/R00 recipients were asked, “Why did you not apply for the K99/R00 award?”. There were six choices of responses: received another research training or career development award (please indicate which one), NIH fellowship recipient, non-NIH fellowship recipient, mentor did not recommend, was not eligible, or other (please explain). Nineteen of these 323 respondents were randomly chosen for a follow-up phone interview to provide additional details as to why these individuals did not choose to apply for the award, and how this in turn may have impacted their careers.

## Results

From FY2007 to FY2022, NCI received 2764 K99/R00 applications in response to the Parent NIH K99/R00 Program Announcements and made 512 awards. The annual NCI K99/R00 success rates ranged from 12.3% (FY2007) to 32.1% (FY2016) with an average of 20.8% over 16 years. The overall success rate for resubmitted applications was 35.9%. Resubmitted applications comprised 20.5% of the overall application pool and 39.6% of all K99 awards (data not shown).

**Fig. 1:**
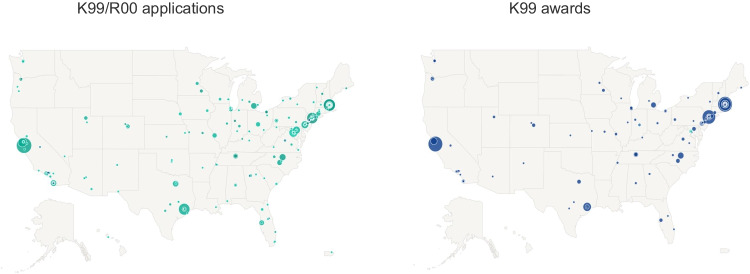
Geographic distribution of FY2007 to FY2020 NCI K99/R00 applications (left panel) and awards (right panel). The size of the circles represents the number of application or awards, respectively

NCI K99/R00 applications were submitted from 225 institutions in 44 US states and territories and were awarded to 108 domestic institutions in 32 states (Fig. 1). We observed significant variability in the organization-specific award rate for organizations that submitted at least 20 NCI K99/R00 applications between FY2007 and FY2017, ranging from 5.7 to 50%. The average award rate of these institutions was 19.7% (data not shown).

NCI K99/R00 applications were reviewed by scientific peer review groups convened by NCI, predominately the Transition to Independence Study Section (NCI-I; https://public.era.nih.gov/pubroster/standingCommitteRoster.era?CID=102233). Reviewers evaluated the K99/R00 applications based on five review criteria: Candidate; Career Development Plan/Career Goals and Objectives (CDP); Research Plan; Mentor(s), Co-Mentor(s), Consultant(s), Collaborator(s) (Mentor); and Environment & Institutional Commitment to the Candidate (Environment) (https://grants.nih.gov/grants/peer/critiques/k.htm). Reviewers assigned overall impact scores that reflected their assessment of the likelihood that the proposed career development and research plan will enhance the candidate’s potential for a productive, independent scientific cancer research career (for applications assigned to NCI). In addition, reviewers evaluated the candidate’s potential for obtaining a tenure-track or equivalent faculty position and developing an independent research program that will make important contributions to NCI’s mission. Applications that were not discussed at the review meeting (typically the lower 50% of the applications based on preliminary scores) received no final numerical overall impact scores and instead received the Not Discussed (ND) designation. The overall impact score is not an average of the individual criterion scores. The final overall impact score is the average of all scores from reviewers who were eligible to vote on that application and multiplied by 10. Criterion scores are only given by the assigned reviewers. Nonetheless, criterion scores can help applicants and NCI staff better understand reviewers’ views on the major strengths and weaknesses of the applications. NCI K99/R00 funding decisions are largely driven by the overall impact score of the applications.

To better understand how reviewers evaluate NCI K99/R00 applications and which elements of the application have the highest impact on the overall impact score, we analyzed the distribution of criterion scores of discussed and not-discussed K99/R00 applications. In addition, we assessed the correlation of criterion scores with the final overall impact scores for only the discussed applications. Similar studies for R01 applications demonstrated that all review criteria are related to the overall impact scores and that the approach criterion is the main predictor of review outcomes [4, 5].

**Fig. 2:**
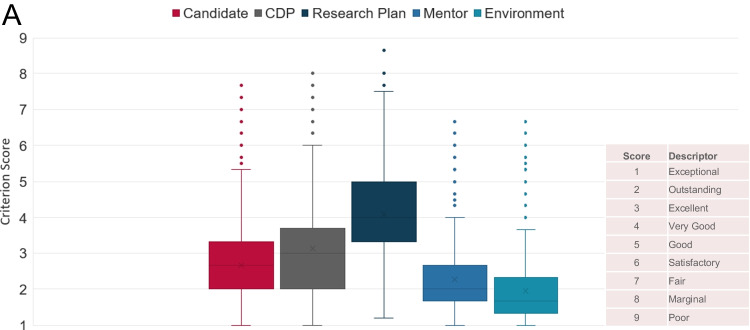

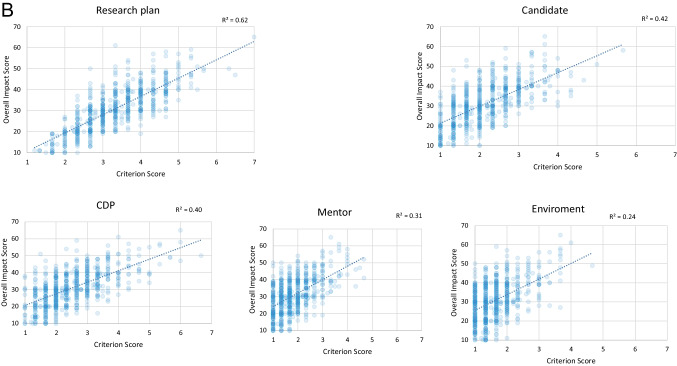
K99/R00 criterion score analysis. Box and Whisker plot of the distribution of criterion scores for K99/R00 applications (discussed and ND) received between FY2010-2017 (2**A**). Linear regression analysis between the overall impact score and criterion scores for FY2010 to FY2017 NCI K99/R00 applications (2**B**). Candidate; Career Development Plan/Career Goals and Objectives (CDP); Research Plan; Mentor(s), Co-Mentor(s), Consultant(s), Collaborator(s) (Mentor); and Environment & Institutional Commitment to the Candidate (Environment)

The distribution of criterion scores for NCI K99/R00 applications (discussed and ND) received between FY2010 and FY2017 was analyzed. While the review criterion scoring range is from 1 (exceptional) to 9 (poor), we noticed that reviewers used a somewhat restricted scoring range for Candidate, CDP, Mentor and Environment with median values below or equal to 3 (Fig. 2A). The Research Plan criterion scores used most of the scoring range and had a median of 4. The CDP and Research Plan criterion scores had the greatest variability with an interquartile range (IRQ) of 1.7. We observed low IRQs of 1 for Mentor and Environment, while the Candidate criterion scores had medium variability with an IRG of 1.3. While all criterion scores were positively correlated with the final overall impact scores, the Research Plan (*r* = 0.78) showed the strongest correlation (Table 1). Environment had the lowest correlation with the overall score (*r* = 0.49). The individual criterion scores were also positively correlated to each other. The strongest correlation between criterion scores was observed between Mentor and Environment (*r* = 0.63). This could indicate that strong mentors have a high likelihood to be associated with strong research institutions and vice versa. These results suggest that the Research Plan was the main review criterion differentiating NCI K99/R00 applications and the criterion scores for Candidate and CDP had some impact while reviewers generally considered Mentor and Environment to be in the Excellent to Exceptional range for most applications.

**Table 1:** Correlation between the overall impact score and criterion scores for FY2010–2017 NCI K99/R00 applications. Candidate; Career Development Plan/Career Goals and Objectives (CDP); Research Plan; Mentor(s), Co-Mentor(s), Consultant(s), Collaborator(s) (Mentor); and Environment & Institutional Commitment to the Candidate (Environment). Not-discussed applications have been excluded

	*Score*	*Candidate*	*CDP*	*Research plan*	*Mentor*	*Environment*
Score	1.00					
Candidate	0.65	1.00				
CDP	0.63	0.50	1.00			
Research plan	0.78	0.46	0.45	1.00		
Mentor	0.55	0.40	0.54	0.42	1.00	
Environment	0.49	0.47	0.50	0.36	0.63	1.00

These results were further supported by linear regression analysis between criterion scores (independent variable) and the final overall impact scores of all discussed applications (dependent variable) (Fig. 2B). The Research Plan was the strongest predictor of the overall impact score with a coefficient of determination (*R*^2^) of 0.62. Candidate and CDP had intermediate predictive strength with *R*^2^ values of 0.42 and 0.40 respectively. The Mentor and Environment criterion scores had the lowest *R*^2^ values of 0.31 and 0.24 respectively. NCI K99/R00 applications with Overall Impact scores of 30 or lower were typically within the fundable range during FY2010–2017 (data not shown). We observed that NCI K99/R00 applications with average Research Plan criterion scores between 1 and 2 had overall impact scores in the 10–30 range and were within the fundable range—independent of the criterion scores for the other review criteria. This was not the case for the other review criteria. Applications that received an average criterion score of 1 for Candidate, CDP, Mentor, or Environment received some overall impact scores outside the fundable range. Our data show the Research Plan criterion was the strongest predictor of the overall impact score and funding outcomes.

**Fig. 3:**
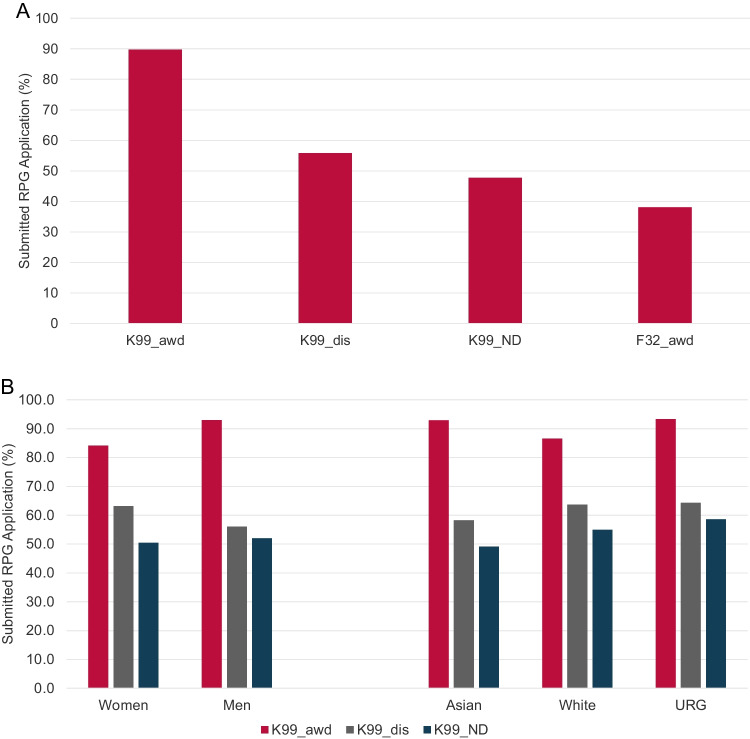
Transition to research independence. Percentage of study cohort PIs who submitted at least one RPG application. NIH RPG application submission rates are used as measure for the PIs’ transition to independence (3**A**). Impact of sex/ gender, race and ethnicity on the transition to research independence (3**B**). K99 awardees (K99_awd), K99/R00 applicants who have not received an NCI K99/R00 award but whose applications received a score during the peer review meeting (K99_dis); NCI K99/R00 applicants whose applications were not discussed and received the ND designation (K99_ND) and F32 awardees. URG: K99/R00 applicants or awardees who self-identified as members of racial or ethnic groups that have been shown to be underrepresented in biomedical research

An important objective of the K99/R00 program is to facilitate the transition of mentored, non-independent scientists to research independence. The submission of NIH Research Project Grants (RPGs) was used as an indicator that a K99/R00 candidate had transitioned from a mentored, postdoctoral training position to an independent academic appointment, since postdoctoral researchers are typically not eligible, based on institutional policies, to submit RPG applications as Principal Investigator. We analyzed whether FY2007 to FY2017 NCI K99 awardees submitted an RPG application as Principal Investigator (PI) or Multiple Principal Investigator (MPI) by the end of FY2022. The major control cohort for this study were NCI Ruth L. Kirschstein National Research Service Award (NRSA) Individual Postdoctoral Fellowship (https://researchtraining.nih.gov/programs/fellowships/F32) awardees. While the objectives of the F32 fellowship are similar to that of the K99 phase of the K99/R00 in that it supports candidates during their mentored postdoctoral training with the goal to enhance their potential to develop into a productive, independent researcher, there are also some differences. The F32 does not have an equivalent to the R00 phase, and we observed that applicants typically submitted F32 applications 2–3 years earlier in their postdoctoral training than K99s (data not shown). For this reason, we analyzed F32 awards using an earlier time window, from FY2005 to FY2015, to adjust for differences in PIs’ career stages at the time of award between the two mechanisms, and to allow similar time for them to transition to research independence. An additional control group were NCI K99/R00 applicants (FY2007–FY2017) who unsuccessfully competed for the NCI K99/R00 award. We divided this group into two sub-groups: (a) K99/R00 applicants who had at least one application discussed during peer review and received an overall impact score (K99_dis) but never received an NCI K99/R00 award and (b) K99/R00 applicants who received the not-discussed designation for all their NCI K99/R00 submissions (K99_ND). We observed the highest transition to research independence rates for the K99 awardees, with 89.8% of PIs in the K99 awardee group submitting at least one RPG application (Fig. 3A). In comparison, only 38.1% of F32 awardees submitted an RPG application. Transition rates of the unfunded K99 applicant control groups, K99_dis and K99_ND, were intermediate with RPG submission rates of 55.9% and 47.7%, respectively. The K99 award did not appear to have a major impact on the time required to transition to research independence. K99 awardees and the control groups K99_dis and F32 awardees required a median 8 years between their PhD and first RPG submission, while K99_ND PIs had a median of 9 years (data not shown).

In order to understand if sex/gender, race, and ethnicity have an impact on the transition of NCI K99/R00 applicants to research independence, we evaluated the RPG submission rates for these groups from FY2007 to FY2017. Women scientist K99 awardees had a somewhat lower RPG submission rate than men with 84.1% compared to 93%, respectively. K99/R00 awardees who self-identified as members of racial or ethnic groups that have been shown to be underrepresented in biomedical research (URG), such as black or African American or Hispanic, had the highest RPG submission rates (93.3%), followed by Asian scientists (92.9%). White K99/R00 awardees had the lowest RPG submission rate of 86.6%. RPG submission rates of the unfunded K99 control groups, K99_dis and K99_ND, were consistently lower than those of K99/R00 awardees (Fig. 3B).

**Fig. 4:**
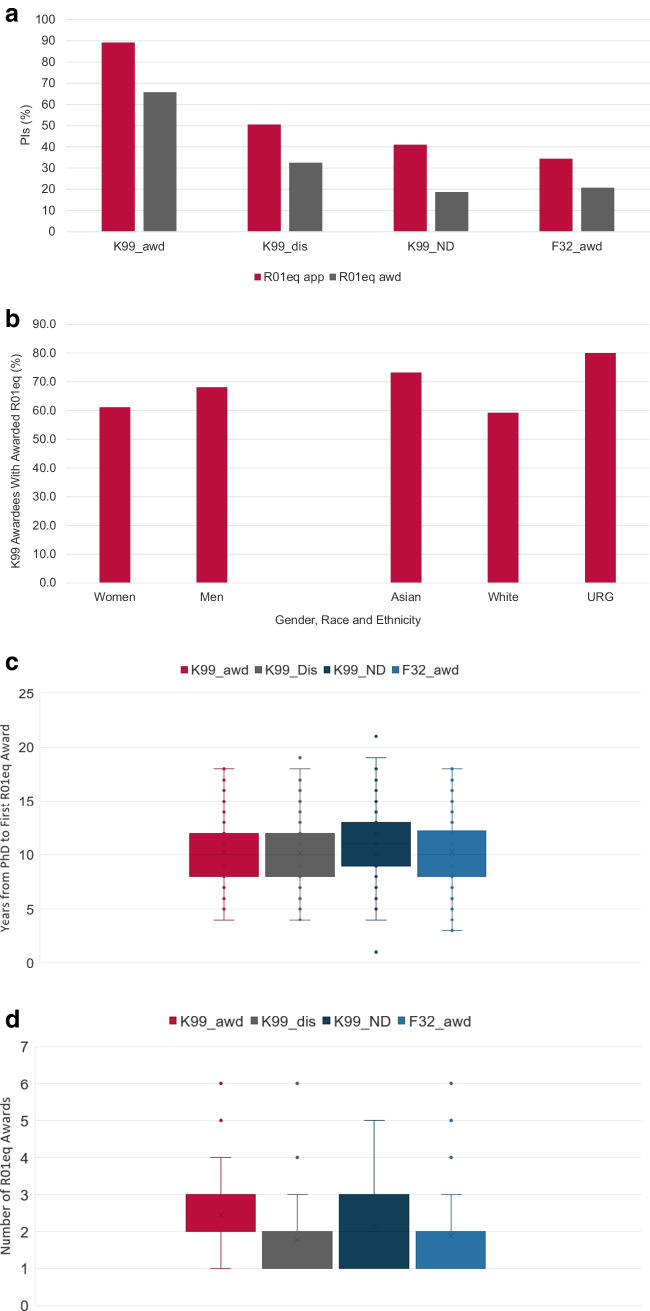
Securing independent R01-equivalent research support. Percentage of study cohort PIs who submitted or received at least one R01-equivalent application or award by the end of FY2022 (4**A**). Impact of sex/ gender, race and ethnicity on securing R01-eq support (4**B**). Time (in years) between PhD graduation and award of the first R01-equivalent grant (4**C**). Box and Whisker plot of the number of R01-equivalent awards within 7 years after obtaining initial R01eq support (4**D**). FY2007 - FY2017 K99 awardees (K99_awd), K99/R00 applicants who have not received an NCI K99/R00 award but whose applications received a score during the peer review meeting (K99_dis); NCI K99/R00 applicants whose applications were not discussed and received the ND designation (K99_ND) and FY2005 – FY2015 F32 awardees. URG: K99/R00 awardees who self-identified as members of racial or ethnic groups that have been shown to be underrepresented in biomedical research

Securing independent funding is a major hurdle for many independent ESIs and is a requirement to establish a productive laboratory. The R01 and equivalent research grants are often seen as foundation for a productive research career. We therefore analyzed if NCI K99 awardees who obtained their K99 award between FY2007 and FY2017 secured R01eq support by the end of FY2022 (Fig. 4A) and compared those results to our control groups. K99 awardees had the highest likelihood to obtain at least one R01eq award during the analysis period with 65.6%, followed by discussed but unfunded K99 applicants (32.4%) and F32 awardees (20.7%). Only 18.6% of not-discussed K99 applicants were awarded at least one R01eq.

Sex/gender, race, and ethnicity had a moderate impact on the K99 to R01eq subsequent award rate. The percentage of K99 awardees who secured R01eq support was 61.1% for women and 68.2% men. When analyzing the impact of race and ethnicity, we observed the highest K99 to R01eq conversion rates for URG K99 awardees with 80% followed by Asian investigators (73.2%) while White investigators had the lowest K-to-R conversion rate of 59.2% (Fig. 4B).

While the K99 award enhanced the likelihood of PIs securing R01eq support, it had no impact on the time required to obtain the first R01eq (Fig. 4C). K99 awardees needed on average 10 years between obtaining their PhD and their first R01eq award. PIs in the control groups needed similar times with 10 years for K99_dis and F32 awardees and 11 years for K99_ND.

Our results suggest that the K99 award enhanced the likelihood of PIs to secure initial R01eq support. Because continuous research support is required to maintain a productive research program, we analyzed if the K99 award affected the PIs’ ability to secure additional R01eq funding. For this study, we evaluated the total number of R01eq awards (counted by distinct base project) that PIs received, within 7 years after their first R01eq award (Fig. 4D). K99 awardees secured on average 2.5 R01eq awards (including the initial R01eq award), while K99_dis, K99_ND PIs, and F32 awardees obtained an average of 1.8, 2.1, and 1.9 R01eq awards respectively.

**Fig. 5:**
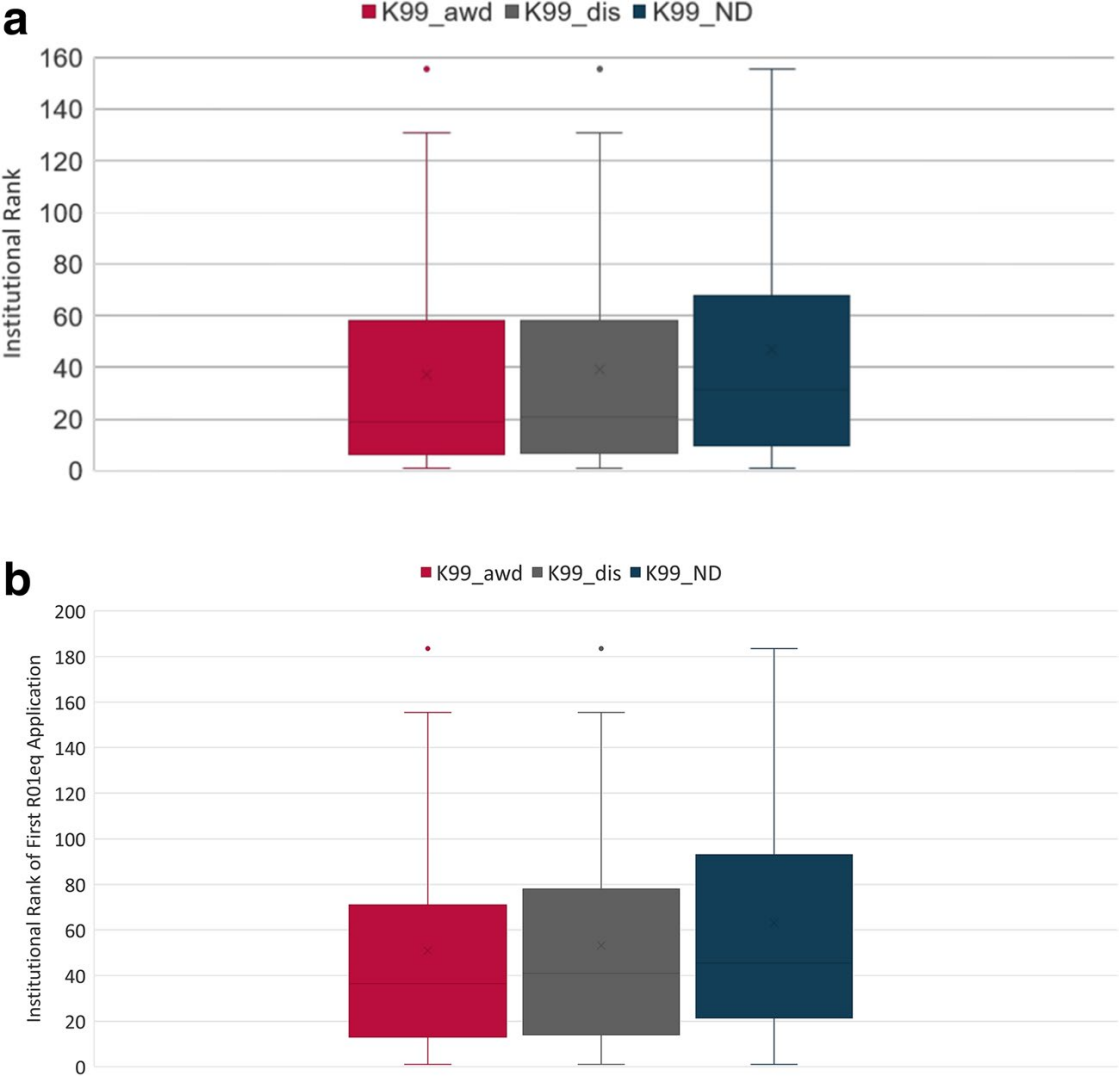
Impact of the institutional cancer resource intensity. Institutional rank at the time of K99/R00 submission (5**A**) and first R01-equivalent submission (5**B**). The institutional ranking system that is based on number of competing NCI R01e awards received by an institution between FY2017-FY2021 as measure of an institution’s cancer research resource strengths. The range of this ranking system is from 1 (162 R01eq awards) to 245 (1R01eq award). K99 awardees (K99_awd), K99/R00 applicants who have not received an NCI K99/R00 award but whose applications received a score during the peer review meeting (K99_dis); NCI K99/R00 applicants whose applications were not discussed and received the ND designation (K99_ND)

The choice of the postdoctoral research institutions can have a significant impact on the research productivity of early-career scientists [6]. It has been demonstrated that institutional prestige predicts early-career productivity. In addition, the prestige of the institution can impact the likelihood of faculty to obtain grant support. We therefore analyzed the impact of the cancer research resource-intensity of the postdoctoral research institution on review outcome (awarded, discussed, or not-discussed). For this study, we used an institutional ranking system that is based on number of competing NCI R01eq awards received by an institution between FY2017 and FY2021 as measure of an institution’s cancer research resource-strength. The range of this ranking system is from 1 (162 R01eq awards) to 245 (1 R01eq award). Fifty percent of R01eq awards went to the top 32 institutions (data not shown). We observed that the median institutional rank for the K99 awardee and K99_dis cohort PIs was similar with a rank of 19 and 21, respectively (Fig. 5A). However, the median rank for not-discussed applicants was 31.5, suggesting that K99/R00 applicants from more resource-intensive institutions have a higher likelihood to be in the upper half of the pool of peer-reviewed applications and to receive funding (Fig. 5A).

Next, we analyzed the institutional rank of the NCI K99/R00 applicants who have successfully transitioned to research independence and submitted an NIH R01eq application (Fig. 5B). The R01eq institutions were less resource intensive than the K99 institutions for all cohorts with a median rank of 36.5, 41, and 45.5 for K99 awardees, K99_dis, and K99_ND cohorts, respectively. However, former K99 postdoctoral scholars appear to have moved to the most resource intensive research institutions of all K99 cohorts for their first independent research positions.

**Fig. 6:**
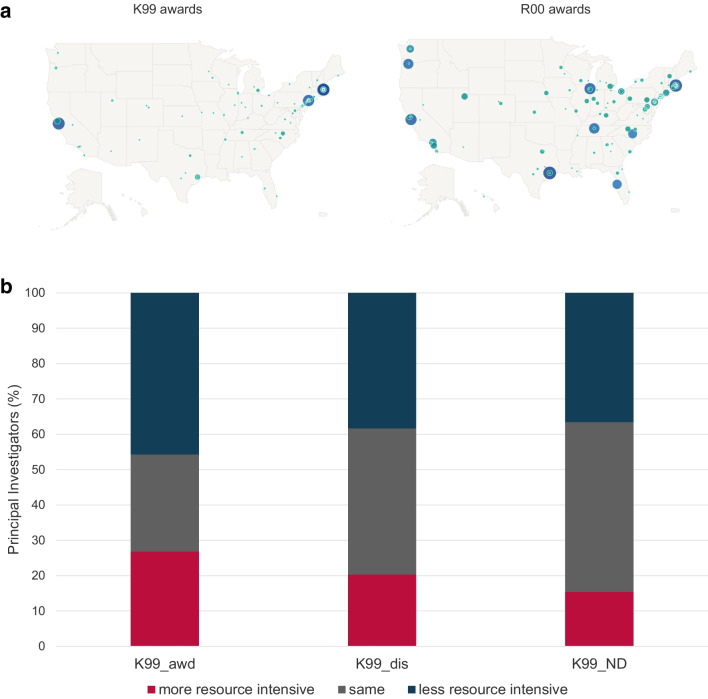

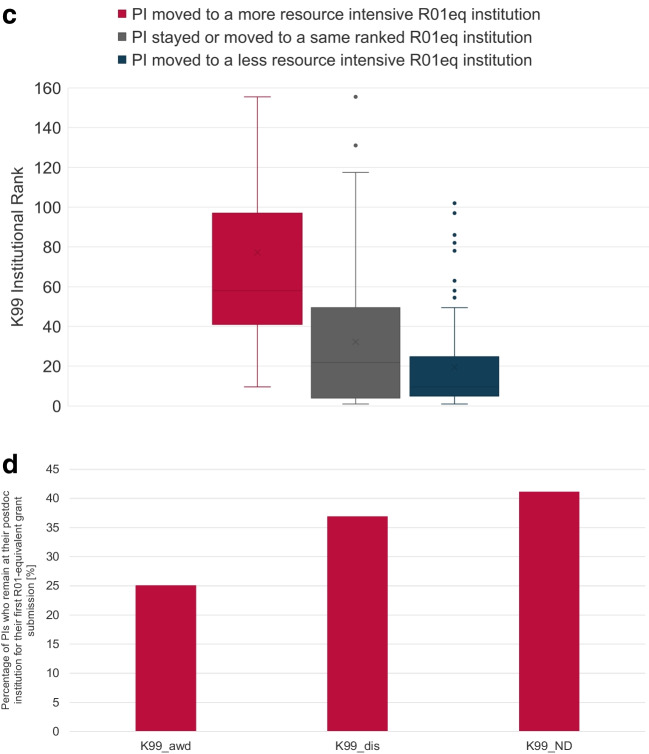
Transition to research independence. Map with the geographic distribution of FY2007 to FY2020 NCI K99 and R00 awards. The size of the circles represents the number of awards (6**A**). Percentage of Investigators who move to a more resource intensive, same or less resource ranked institution when they submit their first R01-equivalent application (6**B**). Box and Whisker plot of the K99 institutional rank of K99/R00 awardees who moved to a more resource intensive, same, or less resource intensive ranked institution when they transitioned to research independence and submitted their first R01eq award (6**C**). Percentage of PIs who remain at their postdoc institution for their first R01-equivalent grant submission (6**D**). K99 awardees (K99_AWD), K99/R00 applicants who have not received an NCI K99/R00 award but whose applications received a score during the peer review meeting (K99_dis); NCI K99/R00 applicants whose applications were not discussed and received the ND designation (K99_ND) and F32 awardees

NCI K99/R00 awardees are encouraged, but not required, to move to a new institution when they transition to research independence and activate the R00 phase of the award. Figure 6A shows the geographic distribution of FY2007 to FY2020 NCI K99 and R00 awards. To better understand the dynamics of the K99 to R00 transition, we analyzed the percentage of K99 cohort PIs who moved to a lower, same, or higher ranked institution when they transitioned to research independence and submitted their first R01eq application (Fig. 6B). We observed that the K99 awardee cohort had the lowest percentage of PIs who remained at an institution with the same institutional rank and the highest percentage of PIs (26.8%) who moved to a more resource-intensive institution. Not-discussed K99 applicants had the highest number of PIs who remained at an institution with identical rank (48%) and the lowest percentage of PIs who transitioned to a more highly funded institution for their independent career (15.4%). Note that identical rank does not necessarily indicate they stayed at the same institution.

K99 awardees who moved to a more resource-intensive organization for their research independence typically came from less resource-intensive institutions with a median rank of 58 while PIs who moved to a less resource-intensive R01eq institution conducted their K99 project at resource-intensive institutions with a median rank of 9.5. PIs who remained at their K99 institution for their independence or moved to a same ranked organization had a median institutional rank of their K99 organization of 21.75 (Fig. 6C).

Since two or more institutions could have an identical rank when they received an identical number of NCI R01eq applications between FY2017 and FY2021, we also determined the percentage of K99 PIs who remained at their K99 institution when they submitted their first R01eq application (Fig. 6D). K99 awardees had the lowest likelihood to remain at their postdoctoral institution for their research independence (25.1%), followed by discussed (36.9%), and not-discussed applicants (41.1%).

These results suggest that discerning the impact of the K99/R00 award on the PI’s selection of the institution for their first independent position is not straightforward. This is in part because our simple ranking system presents an incomplete picture. PIs select their positions from a limited number of options and take into account a host of professional and personal factors in the decision. Therefore, to achieve a better understanding of the benefits and potential limitations of the NCI K99/R00 award, a survey was conducted with both NCI K99/R00 awardees and investigators who chose not to submit an NCI K99/R00 application.

Of 193 K99/R00 past awardees who were surveyed, 95% indicated that the award helped them compete for a position as an independent researcher. For 47% of the survey respondents, their mentor recommended they apply for the K99/R00 award and 42.5% had peers who recommended they apply. Some comments from 10% of the respondents included, “I thought it would be good for my career,” “I wanted to stay in academia,” and “it was a requirement for my program.” Other comments included, “self-determined,” “I felt I was ready,” and “eager to be a PI.” Follow-up phone interviews with past awardees corroborated the written survey responses where 100% of the interviewees indicated the K99/R00 award helped them compete for a position as an independent researcher. It was stated that the award was “pivotal in helping secure interviews and offers from (their) top choice institutions,” as well as making them “highly sought after” by more than one institution during their job search. Having “peace of mind” and the “ability to get their lab started and hire postdocs” was also mentioned as a benefit that would not have been possible without the K99/R00 award. All “highly recommended” their postdocs submit a K99/R00 application.

When individuals who had not applied for the K99/R00 award were asked why, 45.5% indicated they had received another award, 30.9% stated their mentor did not recommend applying, and 31% were not eligible due to time restrictions for eligibility and the fact that the MD training path does not align with the eligibility window for the K99/R00 award and aligns better with the K08 funding opportunity. Select phone interviews were conducted to better understand why individuals never submitted a K99/R00 application. Half of the respondents said they were not eligible, some due to the eligibility window, and some who were MDs that felt there were other awards that better aligned with their career goals. The other 50% were split equally between those who already had other funding, and those whose mentors did not recommend they apply. It was stated that their mentors “did not support trainees applying for grants” or the “mentor said I wouldn’t be competitive.” When asked if they would rethink their decision to apply, 75% said yes, they would, and all felt that the K99/R00 is advantageous in helping secure an independent research position (MDs’ responses excluded). Importantly, all respondents also said they would recommend their postdocs apply for the K99/R00 award.

**Fig. 7:**
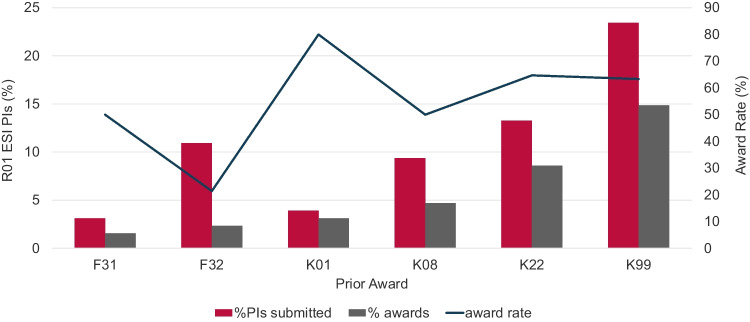
Is prior NIH fellowship or career development grant support crucial for NCI ESIs? Percentage of NCI Early-Stage Investigator (ESI) R01-equivalent awardees who submitted (red columns) or received (gray columns) prior NRSA fellowship or NIH career development support. Line: Fellowship or K-award specific award rate for ESIs

While NIH developed the K99/R00 mechanism to be a conduit from postdoc to independence, some members of the research community have raised concerns that having a K99/R00 award is perceived as a requirement for achieving this transition [7]. Therefore, we asked if prior NRSA fellowship or NIH K award is a prerequisite for becoming an NCI ESI R01 awardee in FY2020–FY2021. We observed that only 14% of NCI ESI R01 awardees submitted an NRSA fellowship application and 50% submitted a K award application, and only 4% of NCI ESI R01 awardees received an NRSA fellowship and 31% received an NIH K award (Fig. 7). The low fellowship application numbers could reflect the fact that the fellowships analyzed require US citizenship or permanent residency for award. The K99/R00 mechanism had the highest number of applications and awards among NCI R01 ESIs of 23.4% and 14.8%, respectively. The K99/R00 award is also the only NIH career development funding opportunity that has no US citizenship or permanent residency requirement. Interestingly, although these data show that a prior F or K award is not required to become an NCI ESI R01 awardee, the award rate, calculated as the percentage of the number of PIs who submitted at least one application and the number of PIs who have received an award, is unexpectedly high for NCI R01 ESIs ranging from 50% for K08s to 80% for the K01 mechanism. These results demonstrate that NCI R01 ESIs represent a group of investigators who were highly competitive for NIH career development awards when they choose to apply.

## Discussion

Our results suggest that the NCI K99/R00 award facilitated the successful transition of postdoctoral scholars to research independence and enhanced the likelihood of K99/R00 awardees to secure subsequent R01-equivalent (R01eq) NIH grant support. NIH RPG application submission rates, which we used as measure for the PIs’ transition to independence, were higher for NCI K99/R00 awardees compared to unfunded K99/R00 applicants and an additional control group, F32 awardees. However, about 10% of K99 awardees did not submit an RPG application. These PIs typically did not activate the R00 phase of their award because they decided to accept a position in industry, moved to an academic institution outside the USA, or were unable to secure an independent tenure-track (or equivalent) academic appointment (data not shown).

It was unexpected that the unfunded NCI K99/R00 control groups (K99_dis and K99-ND) had higher transition rates than F32 awardees. This could indicate that F32 awardees are not fully committed to a career in academia and that new measures may be needed to retain F32 postdoctoral researchers in the academic cancer research fields. Our results may lead one to further examine the overall impact of the NCI F32 award in the academic cancer research workforce development.

The K99/R00 awardees’ sex/gender, race, and ethnicity had only a minimal impact on the PIs’ transition to research independence. The NCI K99/R00 could therefore be an important tool to increase the number of women and individuals in underrepresented groups in the academic cancer research workforce.

NCI K99/R00 awardees also had a higher likelihood than unfunded K99/R00 applicants and F32 awardees to secure at least one R01eq NIH award and obtained the highest median number of R01eq awards. K99/R00 awardees of both sexes/genders and from all racial and ethnic groups were stronger than those control groups in obtaining at least one R01eq award.

However, the NCI K99/R00 award did not appear to have a major impact on the time required to transition to research independence or to receive the first R01eq award. While the award of the K99/R00 did not delay the transition to independence, it also did not appear to make this transition more “timely” compared to our control groups. NIH revised the K99/R00 eligibility criteria in 2013 for FY2014 K99/R00 submissions, reducing the K99/R00 eligibility window from 5 to 4 years of postdoctoral research training experience at the time of application. Initial studies showed that this change led to a 1-year reduction of the time required to secure R01eq support (data not shown).

Our results regarding the impact of the NCI K99/R00 award were confirmed by an online and follow-up phone survey of K99/R00 awardees and non-applicants. With 100% agreement, the survey respondents indicated that receiving a K99/R00 award was influential in helping them transition to research independence. The survey of former K99/R00 awardees and non-awardees highlighted several common themes. Nearly all the K99/R00 awardees felt that the award not only helped them get an interview for a tenure track assistant professor position, but that they were offered more interviews, and received job offers from their preferred institutions. All K99/R00 awardees who now lead an independent lab would recommend their postdoctoral fellows apply for the K99/R00 award.

The survey of K99/R00 non-applicants highlighted the 4-year eligibility window as major hurdle. This is expected; however, it does not capture the undue stress that is felt among many postdoc scholars who experience longer postdocs due to several factors including, but not limited to, manuscripts taking longer to publish, challenges faced in their personal lives, and delayed visa issuance for non-US citizens (https://acd.od.nih.gov/working-groups/postdocs.html). It is important to note that NIH and NCI offer flexibility by extending the eligibility window for life events such as childbirth or caregiving responsibilities. However, all the non-applicants felt that the K99/R00 award would have been helpful and advantageous to their careers as cancer researchers. This group also stated they would rethink their decisions to apply if they had the opportunity. These findings line up with the information from K99/R00 awardees, that this award was beneficial to achieving research independence. Based on the results, an extension of the K99/R00 eligibility window to 6 years of postdoctoral research experience, mirroring the NCI K22, could address these concerns.

Postdoctoral researchers from a diverse range of institutions have submitted K99/R00 applications to NCI. The institutions with the highest application numbers are typically well-established academic organizations with strong cancer research infrastructure and capacity. However, while NCI K99/R00 applicants from some of these institutions experienced high success rates, others did not. The disparity among these otherwise similarly strong research organizations may reflect differences in their commitment in training, mentoring, and supporting the transition to research independence of postdoctoral researchers or could reflect differences in strength of the infrastructure at the organizations to guide submission of NIH grant applications.

The choice of the postdoctoral institution is important for the postdoctoral scholar’s career development. We observed that K99 awardees are predominantly at highly funded institutions with 50% of awardees are at the top 32 institutions based on their NCI R01 research funding. In addition, the K99/R00 award appeared to give postdoctoral researchers flexibility with their choice of institution for their transition to research independence. Among K99/R00 applicant cohorts, K99/R00 awardees had the lowest percentage of PIs who remained at their postdoctoral institution for their independent research career and the highest percentage of PIs who moved to a more resource intensive institution. We noticed that K99 awardees from less resource intensive institutions moved to more highly funded research organizations when they transitioned to independence, likely because the K99 award was an asset to them in their competition for independent research position. However, we also saw that K99/R00 awardees had the highest likelihood among the K99/R00 applicant cohorts to move to an institution with a higher rank (i.e., lower NCI research funding). This is likely caused by the fact that many K99 awardees are already at one of the top funded research institutions and they decided to move to another top-tier institution that has somewhat less NCI funding or that a less resource-intensive institution provided a better research environment for their proposed R00 project, and because there is a limit to the number of faculty positions at any tier. In addition, the institutional ranking system used in this study is based on NCI-specific research funding and is a one-sided and approximate of the quality of an institution which does not factor in the considerations of a specific transitioning investigator. For example, some independent research institutes can provide an excellent research environment in a particular discipline, despite having secured a relatively low number of R01 awards overall. It might be beneficial to use multiple ranking systems, which are based on prestige [6], total NIH funding or funding levels per Principal Investigator, success rates of tenure, and/or a publication-based system to study the transition dynamics in more detail in the future.

Our analysis of the K99/R00 review criteria provided important insights into the review process and showed that while all review criteria scores are positively correlated with the overall impact score, the Research Plan criterion is the strongest predictor of the overall impact score. Importantly, scored applications that received a criterion score of 2 or better for the Research Plan received an overall impact score of 30 or better and were typically within the fundable range. No other criterion score had a comparable impact on the funding outcomes. Therefore, the Research Plan appears to be the major driver for the outcomes of NCI K99/R00 review and funding. This finding is similar to results of an analysis of R01 research grant applications, where the Approach criterion score had the strongest correlation with the overall impact score [4]. This could indicate that study section members evaluate K99/R00 applications similar to the way they review R01 and other research project grant applications. However, since the overall goal of the K99/R00 is workforce development, it might be advantageous to consider redefining the K99/R00 review criteria to encourage reviewers to place more emphasis on the overall potential of the K99/R00 applicant and to focus less on the research plan alone. This would follow suit for the upcoming changes to simplify the framework for the peer review of the majority of competing RPG applications and improving NRSA fellowship review (https://grants.nih.gov/policy/peer/index.htm). The observation that the Institutional Environment is a poor predictor for the overall impact score of K99/R00 applications is somewhat surprising since our results indicate that the strength of the postdoctoral research institution has an impact on review outcomes. This could indicate that the current K99/R00 review criteria for Environment & Institutional Commitment to the Candidate do not allow reviewers to adequately evaluate the strength of the K99 institution.

Overall, the NCI K99/R00 program appears to meet the goals of the K99/R00 career development award and is an important component in NCI’s work to develop and support the next generation of independent cancer researchers. However, neither K99/R00 support nor other NIH career development awards or NRSA fellowships are required to become a successful independent early-stage NCI investigator with substantial NIH funding. While the NCI K99/R00 can enhance the likelihood of postdoctoral researchers to transition to research independence and to secure R01 or equivalent funding, K99/R00 support is not a prerequisite to become a successful investigator, nor does it accelerate the time to research independence. Our results suggest that the NCI K99/R00 mechanism is an important program for retaining postdoctoral researchers in the fields of cancer research and to increasing the percentage of women and promoting diversity of the cancer research workforce. Offering more flexibility by expanding the eligibility window for K99/R00 applicants from 4 to 6 years of postdoctoral research experience could bolster the program to support more promising postdoctoral scholars who have the potential to transition to cancer research independence.

## Data Availability

Public NIH grant records may be downloaded from the NIH RePORTER website (https://projectreporter.nih.gov/). Under the Freedom of Information Act (FOIA), 5 U.S.C. 552, individuals may submit a formal request to obtain information on funded biomedical research grants not publicly available. Inquiries may be directed to the FOIA Coordinator in the Office of Extramural Research at OERFOIA@mail.nih.gov.
