# Correction: Evaluation of the National Cancer Institute (NCI) Pathway to Independence Award (K99/R00) Program

**DOI:** 10.1007/s13187-024-02450-9

**Published:** 2024-05-16

**Authors:** Michael Schmidt, Corinne Boulanger-Espeut, Grace Liou, Nan Ma, Sasha Torres, Susan Cersosimo, Oliver Bogler, Nastaran Zahir

**Affiliations:** 1 National Cancer Institute, National Institutes of Health, Rockville, MD 20850 USA; 2 Digital Science, Inc, Cambridge, MA 02139 USA

**Correction: Journal of Cancer Education**

10.1007/s13187-024-02420-1

The original version of this article contained mistakes.

Fig. 1: left map is cut off in the right corner

Fig. 2: key at top of graph says Criterion Score instead of Environment

Fig. 4c and 4d: key at top of graph says Y-axis title instead of F32.

Fig. 5a: key at top of graph says Y-axis title instead of K99_ND

Herewith are the updated figures.


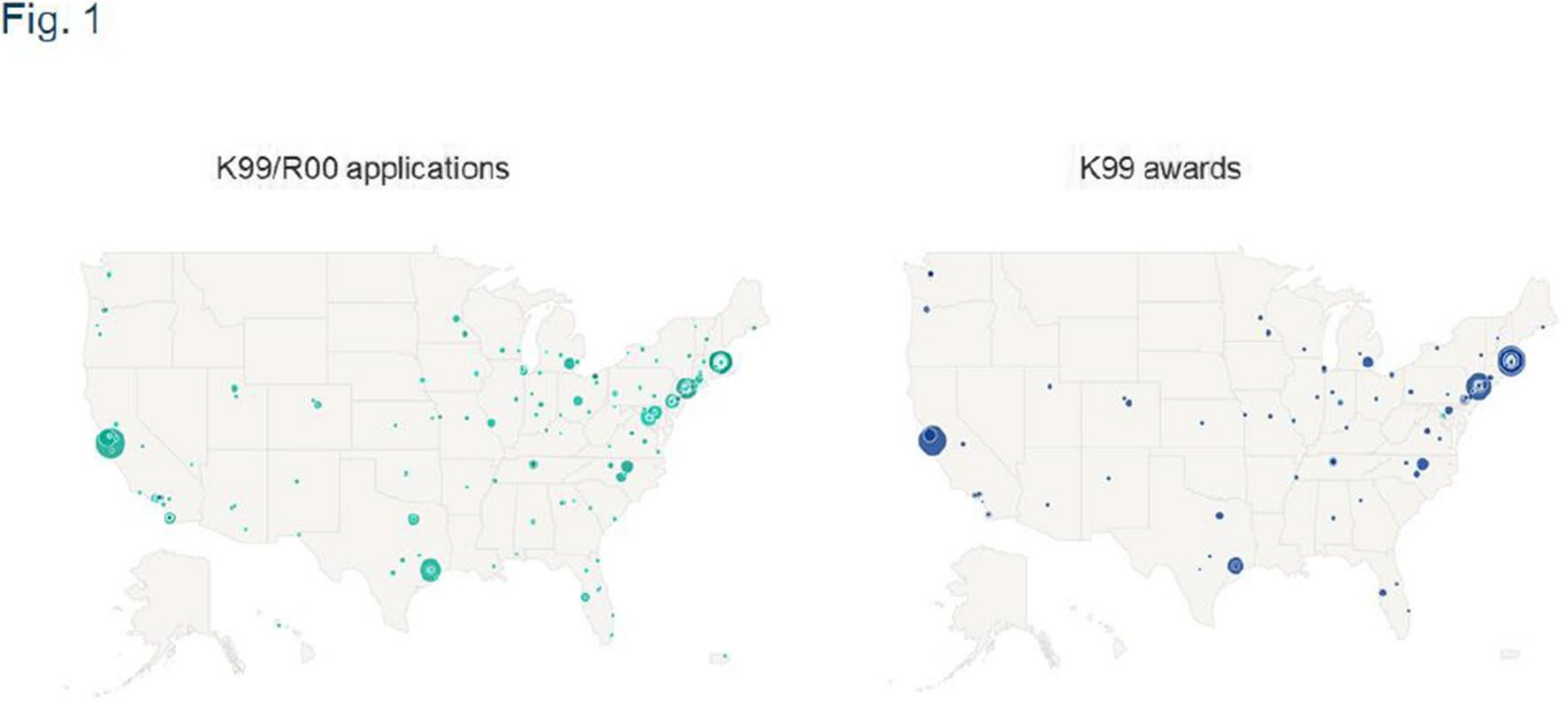



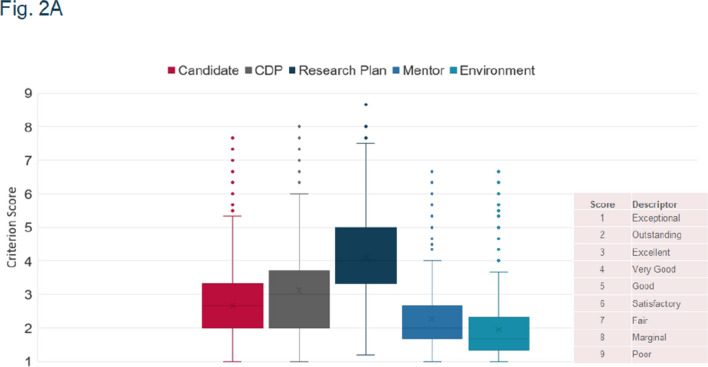



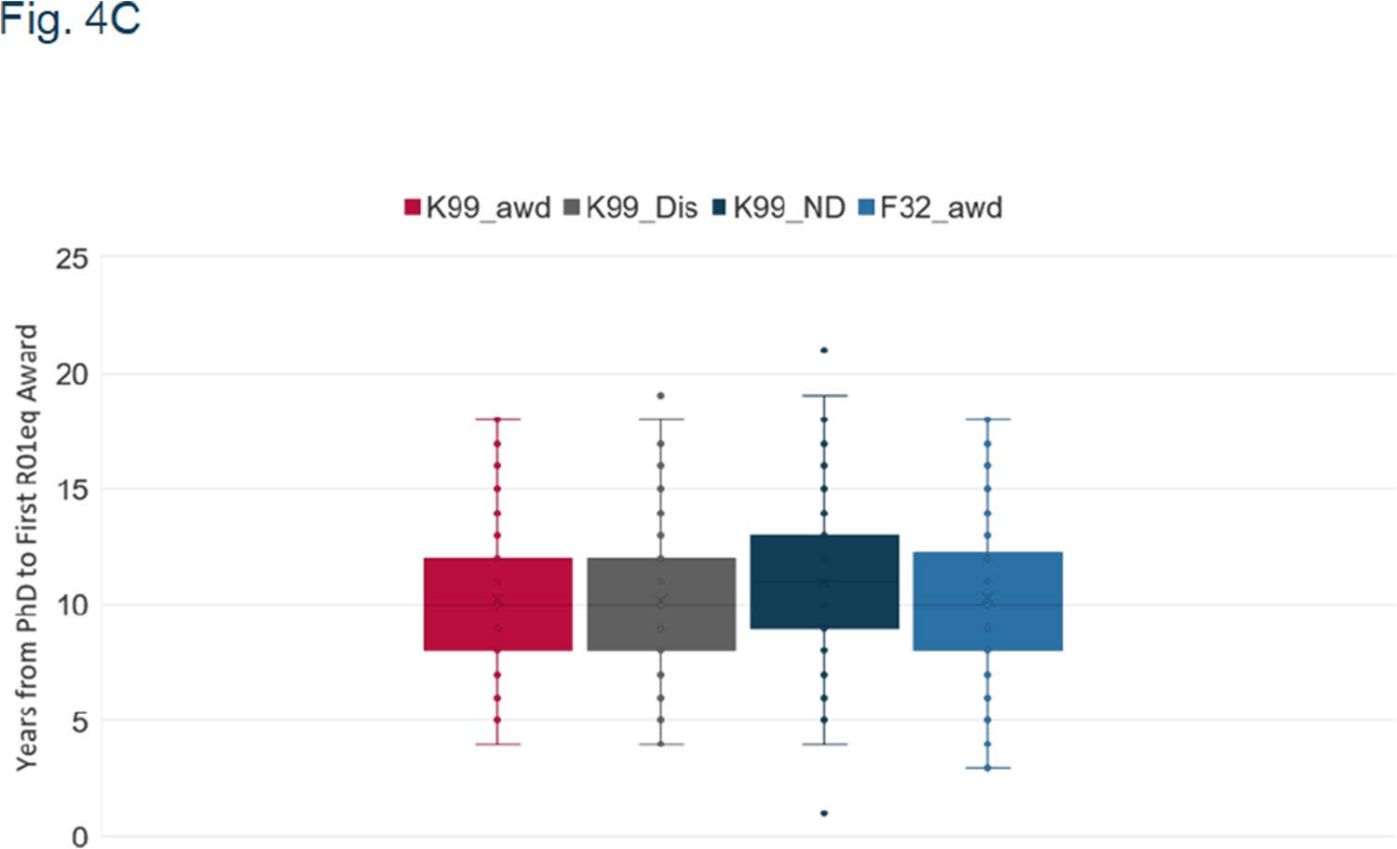
 
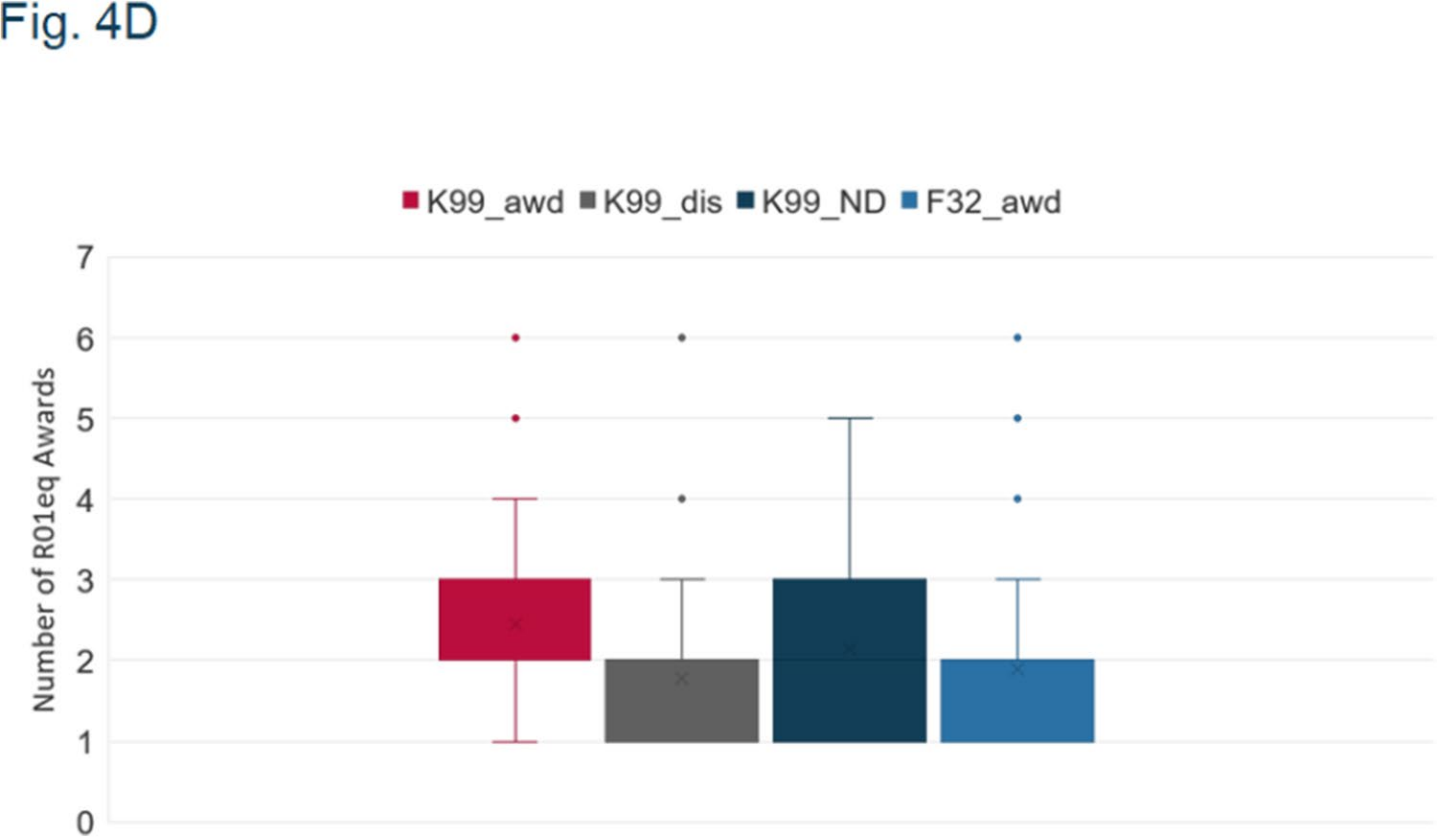



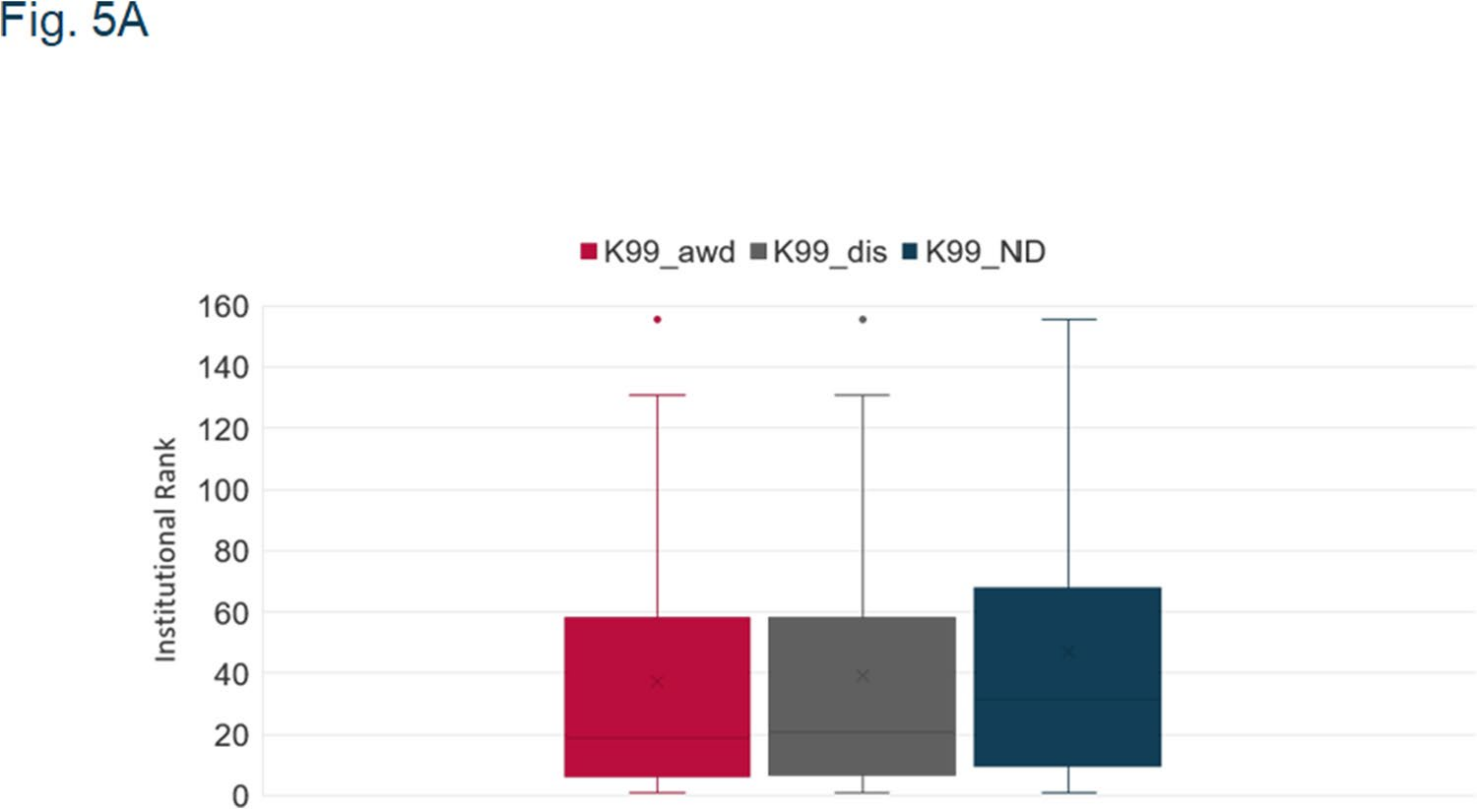


The original article has been updated.

